# Mitigating Sarcopenia with Diet and Exercise

**DOI:** 10.3390/ijerph20176652

**Published:** 2023-08-25

**Authors:** Alex Shefflette, Neel Patel, John Caruso

**Affiliations:** Exercise Physiology Program, University of Louisville, Louisville, KY 40292, USA; alex.shefflette@louisville.edu (A.S.); neel.patel.2@louisville.edu (N.P.)

**Keywords:** disuse atrophy, dietary supplementation, exercise

## Abstract

Sarcopenia is the loss of muscle mass and function from aging, inactivity, or disuse. It is a comorbidity to numerous conditions that exacerbates their severity and adversely impacts activities of daily living. While sarcopenia now receives more attention from the medical community, people with sarcopenia as a comorbidity nevertheless still sometimes receives less attention than other presenting diseases or conditions. Inevitable doctors’ visits or hospital stays for those with sarcopenia as a comorbidity have far higher healthcare costs than those without this condition, which imposes a greater financial burden on the medical insurance and healthcare industries. This review offers information and guidance on this topic. Treatments for sarcopenia include dietary, exercise, and pharmacological interventions. Yet, the latter treatment is only recommended in extreme cases as it may evoke numerous side effects and has little support in the scientific literature. Currently, a more holistic approach, with an emphasis on lifestyle modification, to reduce the likelihood of sarcopenia is examined. The current review discusses dietary and exercise interventions to limit the occurrence and severity of sarcopenia. References cited in this review conformed to the Declaration of Helsinki requirements for the use of human research subjects. Most of this review’s references (~97%) came from a PubMed search that spanned from 1997 to 2023. Search terms included “sarcopenia” OR “muscle wasting” OR “geriatrics”; OR “ageing”; and AND “diet” OR “exercise”. In addition, papers relevant or supportive of the topic as well as those considered seminal were included in the review. Over 96% of the references were peer-reviewed articles.

## 1. Introduction

Sarcopenia, a term coined by Rosenberg in 1989, was a skeletal muscle-wasting disease that went unnoticed or untreated due to the lack of diagnosis or minimal attention it received [1]. It was finally acknowledged as a disease in 2016 when the World Health Organization released an International Classification of Diseases (ICD; code: M62.84) for sarcopenia [2]. It is primarily associated with advanced aging as 36.5% of adults aged ≥ 60 years have sarcopenia [1]. But as physically inactive lifestyles become more prevalent, it is more common at younger ages [3,4,5,6,7]. Sarcopenia is linked to both type II diabetes and obesity. For instance, since 80% of glucose’s uptake from the blood is taken up by skeletal muscle under euglycemic conditions, a decline in muscle mass and/or quality reduces blood glucose clearance, leading to hyperglycemia and a higher type II diabetes risk [8]. Myosteatosis, or sarcopenic obesity, is excessive fat deposition in muscles [9,10,11]. It causes a positive energy balance and undermines muscle quality [9]. Myosteatosis reduces muscle strength, mobility, and a person’s mortality and may increase their risk of underlying diseases [9,10]. Sarcopenia increases the risk of physical disability as well as chronic cardiovascular and respiratory diseases [3,12,13,14]. Primary sarcopenia, the version observed in older persons, is a multifactorial disuse condition that entails perturbations to myostatin, inflammatory cytokines, and mitochondrial function as well as a loss of satellite cells, motor neurons, and anabolic hormones [14,15]. Primary sarcopenia has no specific cause other than ageing [3]. However, secondary sarcopenia, the type observed in young people primarily due to disuse/inactivity, may also result from systemic disease or malnutrition [3].

Several organizations independently offered a working definition of sarcopenia [16,17,18]. The 2018 European Working Group on Sarcopenia in Older People (EWGSOP) revised the definition to not just specifically pertain to muscle atrophy but to include muscle strength and physical performance deficits [3,5]. The EWGSOP determined sarcopenia’s three diagnosing criteria in order of importance which were a loss of muscular strength, decrements in muscular quantity and/or quality (atrophy and/or muscular disuse), and low physical performance [3,5]. Since the 2019 Asian Working Group for Sarcopenia (AWGS) termed it an “age-related” loss of muscle mass, strength, and/or physical performance with an age cutoff of at least 65 years [16], the EWGOSP redefined it to encompass both muscle quantity and quality, of which the latter relates to its force generating capacity [3,7,19,20]. Screening for sarcopenia uses the SARC-F questionnaire [21]. Yet, to diagnose sarcopenia, strength tests’ strength entail hand-grip dynamometry or resistance exercise equipment. Physical performance tests to identify sarcopenia include the Timed-Up and Go (TUG) or 6-min walk field tests [5,22,23]. Accurate muscle quantity measurements, the costliest to diagnose, use either dual-energy X-ray absorptiometry (DXA), magnetic resonance imaging (MRI), computed tomography (CT), or appendicular skeletal muscle mass (ASMM) via bioelectrical impedance [4,5,7,20,24].

In terms of temporal changes, sarcopenia may be subdivided into acute (<6 months) and chronic (≥6 months) conditions [3]. Sarcopenia is characterized by a 3–8% loss in lean muscle mass per decade after 30 years of age [20,24,25,26]. Others state that it is present if the absolute volume of a person’s muscle mass is >2 standard deviations below a normal z-score value [3,5]. The progressive decline in skeletal muscle mass significantly accelerates after 70 years of age (approximately 15% per decade). Sarcopenia affects 30% of individuals over 60 years of age and more than 50% of those over 80 years [27]. Others suggest that it affects 5–13% and 11–50% of older adults aged 60–70 years and above 80 years of age, respectively [28,29]. It was identified in roughly half of male nursing home patients and may serve as a gateway disorder that increases the prevalence of conditions that undermine a person’s health [14]. With a world population whose average life span is increasing, sarcopenia is more common and is an added financial burden to the medical insurance and healthcare industries. Each year, the United States spends roughly 40 billion USD in hospital costs for patients with sarcopenia, which could be reduced with proper preventative measures. They include diet and exercise; each preventative measure should first be approved by a patient’s family physician before they are implemented. Prior to discussing treatments to mitigate sarcopenia, it is important to identify how poor diet and lifestyle (i.e., exercise habits) choices contribute to this condition.

## 2. Dietary Causes: Energy, Nutrients, and Nutritional Supplementation

### 2.1. Energy

In terms of energy, sarcopenia results from a negative caloric state or a lack of specific macronutrients in a person’s diet [7]. Older populations are susceptible to primary sarcopenia due to malnutrition. In support of this statement, a recent study assessed intermuscular adipose tissue (IMAT) and skeletal muscle radiodensity (SMD) via a CT scan in relation to malnutrition in older hospital patients [7]. As compared to older patients with normal nutrition, the malnourished had significantly lower SMD. IMAT was positively associated with malnutrition though this was not statistically significant [7]. Yet, among young people, such as those with eating disorders or non-age-related diseases, age was not a factor for secondary sarcopenia. Growing evidence supports changes to basic nutritional guidelines to combat muscle wasting, particularly in reducing negative caloric balances [7,30,31,32]. Higher energy intakes have long been advocated for many disuse atrophy conditions, among them spaceflight [33,34]. With abrupt increases in metabolic rates, as well as the absence of mechanical loading and weight-bearing forces combined with a suppressed appetite, such rapid changes during spaceflight not only simulate the muscle wasting incurred with sarcopenia but occur at far faster rates [33,34]. While sarcopenic patients incur many of the changes observed with microgravity, spaceflight research advocated for higher energy intakes to limit the severity of body and muscle mass, as well as strength, losses [33,34]. Adopting similar changes in persons with sarcopenia may also mitigate such losses.

Hospitalized older adults (n = 1135) were subdivided into normal and malnutrition groups to identify associations with handgrip strength [7]. With muscle mass quantity and quality indices examined as correlates of handgrip strength, values from older adults with normal nutrition were compared to the malnourished and the latter had significantly less skeletal muscle density and strength [7]. Malnourished women had significantly more IMAT than their normal group counterparts while malnourished men had a significantly lower muscle quality than the normal group [7]. Since low energy intakes yield negative caloric balances in malnourished and sarcopenic patients [7], higher food consumption was encouraged for these groups like they were for astronauts [33,34]. Finally, rates of sarcopenia and poor muscle quality were assessed in young (n = 108, 43 ± 11.7 years) obese adults, a group prone to falls, muscle pain, and bone fractures [4]. This is an issue as sarcopenic obesity leads to comorbidities (diabetes, hypercholesterolemia, and pulmonary diseases) that increase the risk of premature death. Study measurements included DXA scans, handgrip strength, TUG, gait speed, and ASMM adjusted for BMI [4]. Rates of sarcopenia ranged from 11.1–13.9% and it was most common in middle-aged women. Muscle quality was deemed important to functional disability and must be considered in sarcopenia assessments [4]. It was concluded that a need exists to stringently standardize criteria for young adults with sarcopenic obesity as its occurrence varies widely for this population [4]. Its management sees doctors prescribe a reduced-calorie diet high in protein, Ca^2+^, and vitamin D as well as exercise.

### 2.2. Protein

Macronutrients deficient in persons with primary sarcopenia include protein. Compared to young adults, older populations eat less, including less protein [35,36]. In Europe, up to 10% of community-dwelling older adults and 35% of those institutionalized do not meet the estimated average requirement for protein of 0.7 g·kg body mass^−1^·day^−1^, the minimum amount needed to maintain muscle in adults of all ages, while the USRDA for protein is 0.8 g·kg body mass^−1^·day^−1^ [30,36]. This is concerning since older persons have higher protein needs than young adults [37,38]. The imbalance between protein requirements and intake produces net muscle mass losses due to a chronic imbalance between cellular protein synthesis (MPS) and degradation [39]. As a result, older populations are susceptible to sarcopenia’s muscle mass and strength losses [40,41]. Consumption of whole food protein sources easily meets the needs for this macronutrient; however, this is more difficult to achieve in older adults since they eat less [35,36]. While animal-based foods are a prime source of dietary proteins, plant-based foods lack one or more essential amino acids (EAA). Yet, food combining, whereby multiple plant-based foods are present at a given meal, better assures all EAA are provided by a person’s diet. Despite food combining and animal-based foods as excellent high-quality sources, protein is insufficient in the diets of older people.

Traditional dietary recommendations do not meet the protein requirement to abate muscle loss or stimulate hypertrophy in older adults [2]. Older sarcopenia patients may benefit from higher protein intakes not just for the energy it provides but to aid cell growth and repair. Evidence suggests sarcopenia may be abated by higher protein intakes [30,42]. The European Society for Clinical Nutrition and Metabolism (ESPEN) made the following recommendations: (1) the diets of healthy old people should provide at least 1.0–1.2 g protein/kg body mass/day, (2) the older malnourished or those at risk of malnutrition should consume 1.2–1.5 g·kg body mass^−1^·day^−1^, with even higher intakes for those with severe illness or injury, and (3) daily physical activity or exercise for all older people for as long as possible [43]. In the absence of exercise as a person ages, higher protein intakes help achieve a net neutral energy balance and reduce muscle catabolism. Older persons do not have mitigated anabolic responses to high-quality protein meals. Evidence shows that maximal muscle protein synthesis (MPS) occurs with 25–30 g of the macronutrient provided per meal regardless of if the person is young or old [44]. Yet, MPS is blunted in older adults when protein is co-ingested with carbohydrate or when its intake is less than 20 g per meal [44]. Evidence shows that 10 and 20 g servings of EAA did not stimulate MPS in old people to the same degree as in the young [45]. Ageing may impair anabolism in response to a carbohydrate-containing mixed nutrient meal, as shown by ingestion of an amino acid-glucose mixture that saw MPS rise in young but was unchanged in older subjects [44,46]. Subsequent research confirmed these results [47]. In older groups, mixed-nutrient meals may require leucine supplementation to enhance MPS [44]. No evidence exists that co-ingestion of protein and fat negatively or differential effects protein anabolism in the young or old [47,48].

Fortunately, even modest bouts of physical activity may sensitize ageing muscle to create a more anabolic environment. For instance, 45 min of treadmill walking by older persons restored their ability to utilize insulin, which can aid MPS [49]. The ingestion of leucine-rich amino acids stimulated MPS to a similar extent in young and old subjects for the first six hours post-exercise [50], albeit with a delayed response in the elderly. These data offer further evidence of physical activity’s role in restoring or maintaining normal protein anabolic responses in the elderly. Higher amounts of protein appear essential for muscle growth and repair in older persons since ageing reduces responsiveness to low dietary doses (7.5 g) of EAA but higher doses (10–15 g) stimulate MPS at levels comparable to that of younger persons [3,51]. In addition, supplementing mixed-nutrient meals with leucine may enhance MPS in the elderly. Older men (n = 37; 71 ± 4 years) were given a whey protein supplement to examine MPS changes [51]. They first completed lower body strength training workouts and then consumed 0, 10, 20, or 40 g of whey protein isolate. MPS rose by 65% and 90% for those who ingested 20 and 40 g post-workout, respectively, while lower doses led to insignificant changes [51]. Another study noted that young adults needed only 40 g to saturate MPS rates, suggesting whey is a valuable leucine source but older adults should probably ingest a higher dose to obtain comparable benefits [52]. Another study noted that 15 g of EAA given to older patients three times daily produced a net neutral 24-h fractional synthetic rate, which is an MPS index and more evidence higher protein intakes are needed for this population [53].

Recommendations for older adults include consuming 25–30 g of protein per meal and sufficient energy intake, rather than a higher absolute daily increase in protein [44]. The latter approach produces a skewed distribution of protein intake that does not maximize MPS throughout the day [54]. To provide enough protein to abate sarcopenia, dose-response relationships to achieve MPS proposed a per-meal intake of 0.4–0.6 g·kg body mass^−1^·meal^−1^, though some advocated per-meal intakes of 0.8 g·kg body mass^−1^·day^−1^ [54]. In older adults and those with sarcopenia, a net-neutral protein balance requires an even higher dietary intake than previously thought [54]. For instance, Kim et al. noted that to optimally maintain an anabolic response to a mixed food intake, instead of using a dietary protein supplement, older adults need to ingest 1.8 g·kg body mass^−1^·day^−1^ [30]. This is over twice the recommended intake for adults advocated by the USRDA [30,54]. While admittedly a very high protein intake, current recommendations to reduce sarcopenia include greater protein consumption. Other recommendations entail an even distribution of protein throughout the day and a leucine-rich diet since it stimulates MPS [54]. With no crossover, 56 sarcopenic patients, who were at least 65 years of age, ingested 1.2–1.5 g·kg body mass^−1^·day^−1^ of protein daily either through a combination of food, leucine-infused whey protein, and vitamin D supplements or solely through food [55]. ASMM, handgrip strength, gait speed, energy, and macronutrient intakes were measured after 4 and 12 weeks for each group. While the total energy and protein intakes rose in both groups, supplementation created significant differences in protein intake [55]. Compared to the food-only group, supplementation improved gait speed after 12 weeks, particularly for those less than 75 years of age. Supplementation also allowed the sarcopenic elderly to easily meet the study’s protein requirements [55].

Finally, branched-chain amino acids (BCAA) were examined as a possible treatment to reduce muscle wasting and sarcopenia [43]. BCAA leucine positively regulated intracellular signaling pathways to induce MPS [56]. A higher leucine intake was needed to optimally stimulate MPS in older, as compared to younger, adults [52]. In critically ill adults, mixed BCAA supplementation increased MPS [57,58]. In a randomized controlled study with older sarcopenic women, those who exercised and supplemented their diets with leucine increased their leg muscle mass and strength and had faster walking speeds [59]. Moreover, 16 weeks of EAA supplementation in older adults led to greater muscle mass and function without concurrent exercise [60]. Yet, other studies showed that long-term leucine supplementation alone did not increase muscle mass or strength [61,62]. While age-related changes reduce MPS in older adults, such a change can be reversed by higher protein intakes and greater amounts of exercise [63,64]. The remainder of this review’s diet section examines other considerations thought to limit the occurrence of sarcopenia. They include vitamin D, Ca^2+^, and Mg^2+^ as well as beta-hydroxy beta-methylbutyrate (HMB) supplementation. Some studies examined as part of the next section of this review looked at these other considerations concurrent to protein supplementation.

### 2.3. Vitamin D

A focus on protein, especially if obtained from animal products, allows the intake of other micronutrients without resorting to dietary supplements. Such micronutrients include vitamin D, which aids bone remodeling and immune function, each of which are important issues for older adults. Since vitamin D and protein are nutrients common to many foods, research examined their combined effects in sarcopenic patients [65]. A study with older men (n = 49; 73 ± 1 years) had them ingest either a supplement with whey protein, creatine, Ca^2+^, vitamin D, and omega-3 fatty acids or a maltodextrin drink that served as a control with no crossover [66]. The study’s first phase required subjects to ingest beverages twice daily in accordance with their group assignment while phase two entailed their consumption concurrent with exercise three times per week. For the first phase, only the supplemented group had strength increases. While both groups gained strength during phase two, those who received the supplement had significantly higher strength, lean muscle mass, and aerobic capacity gains. A glucose tolerance test revealed that maximal glucose concentrations declined in supplemented patients, suggesting a link between muscle uptake and glucose management. The lean muscle mass and glucose concentration results suggest the supplement benefitted older men with sarcopenia and may lower their risk of type II diabetes [66]. As a result, current dietary therapies advocate for 20 g of whey protein and 800 IU of vitamin D twice daily for those with sarcopenia [14].

A systematic review and meta-analysis showed that concurrent vitamin D and protein supplementation offers strength benefits to those with sarcopenia; yet, disagreement on this topic exists [65,67]. Those who disagree cite a review that stated that no strong evidence existed for concurrent vitamin D and protein supplementation to improve muscle mass and function due to mechanisms underlying vitamin D’s role within skeletal muscle [67]. That same review claims greater benefits occurred in patients who severely lacked the vitamin [67]. Vitamin D deficiency exists in 41.6% of all US adults [68]. Recent vitamin D recommendations rose from 400 IU/day to 600 IU/day for persons less than 70 years of age and from 600 IU/day to 800 IU/day for those who are older [69]. However, these higher dosages are a general guideline and do not account for individual activity levels or medications that interfere with vitamin D metabolism [69,70].

### 2.4. Ca^2+^ and Mg^2+^

Each mineral aids in muscle fiber contraction so it is assumed that a deficiency in either poses a problem to muscle function, particularly among those with sarcopenia [30]. In addition to Ca^2+^’s role of in crossbridge formation, Mg^2+^ facilitates ATPase activation; thus, a deficiency in either would impair muscle function and force output. Ganapathy and Nieves reported that most older persons do not consume enough Ca^2+^ [31]. When provided as a singular dietary supplement, Ca^2+^ had mixed effects on strength gains [31]. Currently, not enough research exists to conclude if Ca^2+^ alone increases strength [31]. When Tieland et al. reviewed dietary guidelines for sarcopenic patients, they concluded that Mg^2+^ supplementation improved exercise performance for intense short-term bouts, presumably due to its role in ATPase activation [71].

Ca^2+^, Mg^2+^, protein, vitamin D, and energy each have specific and vital roles for muscle function and mass preservation to reduce the risk of sarcopenia. A healthy balanced diet rich in whole foods provide each of the nutrients. Yet, some have also proposed dietary supplements. Among the many supplements to reduce the risk of sarcopenia is HMB, which is an over-the-counter product purportedly safe to use [72].

### 2.5. Beta-Hydroxy Beta-Methylbutyrate (HMB)

HMB is naturally produced in the liver through a reversible cascade from the breakdown of leucine [72,73]. Protein kinetics that enhance MPS through HMB supplementation may do so through numerous intracellular mechanisms. Among them are the mTOR anabolic and the PI3/Akt signaling pathways. While mTOR is directly involved in MPS, PI3/Akt relies on phosphorylation for downstream activation of effector organelles, including those which regulate protein catabolism. HMB both increases mTOR activity and reduces proteolysis by blunting protein phosphorylation along the PI3K/Akt pathway to improve intracellular MPS [17,74]. HMB at dosages higher than those produced endogenously reduced muscle wasting in cancer patients [75]. This occurred from reduced proteolysis and a surpression of tumor necrosis factor alpha [74,76]. A review of HMB articles, with its administration concurrent to exercise, showed improvements in muscle mass, strength, and aerobic capacity among trained and untrained subjects [73]. Sarcopenic patients (n = 41; 66–84 years old) ingested 8 g of EAA for 18 months and increased their muscle mass, insulin sensitivity, and insulin-like growth factor 1 over time [76]. HMB administered at 2 g·day^−1^ to bedridden older subjects significantly reduced muscle wasting [77]. A mixture of 3 g HMB, 7.5 g arginine, and 2.25 g lysine given to older subjects increased their protein turnover and lean tissue mass [78]. Since amino acids were suggested to treat muscle wasting, leucine, glutamine, arginine and HMB supplementation were examined for sarcopenia management in geriatric patients [6]. Results suggested leucine and HMB supplementation may be used to treat sarcopenia [6]. Arginine administration alone did not prevent muscle wasting but it was effective when combined with HMB and leucine [6]. It was concluded that no single amino acid prevents sarcopenia but in combination can increase muscle mass and strength in geriatric patients [6]. While these results are encouraging, perhaps HMB is most beneficial for sarcopenic patients when combined with resistance exercise [72,75,76,77,78]. Some sought to approximate an optimal HMB dose for muscle hypertrophy [73]. A dose-response relationship appears to exist for up to 3 g·day^−1^ of HMB when used concurrent to strength training [73]. Thus, HMB supplementation reduces the occurrence and severity of sarcopenia with concurrent resistance exercise.

## 3. Lifestyle Causes: Exercise and Sarcopenia

Lifestyles devoid of exercise contribute to sarcopenia. Adaptations to this lifestyle inevitably include muscle mass and strength losses. The prevalence of this condition increases in the elderly, particularly after the age of 65, as 14.7% of hospitalized patients incur sarcopenia while under medical care [11,79]. Intuitively, reversing an inactive lifestyle with exercise sees those with sarcopenia literally able to treat themselves if implemented at an early time point for their condition. Exercise lowers concentrations of substances like C-reactive protein and interleukin-6 that are associated with inflammation and sarcopenia [80]. Despite these known benefits, only 8.7–10% of older adults (>75 years of age) in the United States participate in resistance exercise which is thought to be ideal for treating sarcopenia [2,81]. Generally, six weeks of resistance exercise is required before noticeable improvements occur [2]. The low exercise participation rates, despite its broad health benefits, underscore the need for evidence-based guidelines and recommendations for older adults to limit their risk of sarcopenia [81]. Properly administered weight training is safe to implement for healthy and frail older adults as well as those with sarcopenia [81].

Specific Adaptations to Imposed Demands (SAID) is a common principle to explain the body’s adaptations to physical activity as well as the occurrence of sarcopenia. SAID refers to the unique and novel pattern of a mechanical loading paradigm and the subsequent adaptations it manifests. The resultant adaptations may have profound impacts on skeletal muscle that may reduce the incidence and severity of sarcopenia [82]. Regarding exercise, SAID typically divides various forms of physical activity into aerobic and resistance exercise. Current recommendations from the American College of Sports Medicine (ACSM) emphasize aerobic training to improve the health of the general population. Yet, a 2020 CDC report claimed just 24.2% of persons over 18 years of age met the general guidelines for both aerobic and resistive exercise [83]. Those who met the guideline for both forms of exercise were more often men (28.3%) than women (20.4%) and numbers decreased with age for both genders [83]. Sadly, 46.3% of the general population failed to meet both aerobic and resistive exercise guidelines [83]. While many benefits from aerobic exercise exist, such as lower reactive oxygen species function that causes cellular apoptosis in primary sarcopenia, there is little evidence that it reduces muscle wasting [15]. While aerobic capacity improvements occur from moderate-to-vigorous-intensity steady-state exercise, resistance training against high loads is an optimal way to treat sarcopenia and improve balance [84,85]. Resistance exercise in healthy older adults, when compared to endurance training, produced larger strength gains with similar improvements in aerobic capacity, suggesting it may be superior to preventing sarcopenia [2,86]. Resistance exercise increases protein anabolism and muscle function while reducing protein degradation in healthy older adults; yet, less is known about its ability to help those with sarcopenia [15,87].

Evidence of an ideal resistance exercise prescription for those with sarcopenia is limited [87]. They should consider basic variables (training frequency, exercise mode, intensity, volume, and rest period duration) as well as a person’s characteristics (baseline fitness, nutrition, genetics, medication usage, and exercise history) that represent their current condition [87]. High-intensity resistance training is feasible and effective for severely frail older people when properly prescribed [88]. Among older people, gains in muscle strength and physical function occurred after six months of a simple daily exercise routine comprising squats, one-legged stands, heel raises, and 20–30 min of walking [89]. Treating older sarcopenic patients with resistance exercise is an effective way to abate muscle mass and strength losses as well as have a more holistic approach to personal wellness [81]. Resistance exercise reduced physical vulnerability as well as improving mobility, independence, disease management, psychological well-being, quality of life, and mortality in older sarcopenic persons [81]. Thus, a shift in exercise prescriptions that emphasize resistance exercise may best reduce sarcopenia [48,64,83,90,91,92,93].

Likely due to strength losses related to their condition, those with sarcopenia have a low tolerance to physical activity and fatigue easily, which complicates their exercise prescriptions. As a result, the number of sets and repetitions needed to induce hypertrophy and strength gains for sarcopenic persons is relatively low and must be individually prescribed. Resistance exercise prescriptions for older adults should last 30–60 min and use the same principles established for younger populations [94,95,96]. Individualized programs are more important for older adults as factors such as health/family histories, injuries, and pre-existing fitness levels must be considered when designing programs [94]. General resistance exercise guidelines for healthy older adults and those with chronic conditions include 2–3 workouts weekly against moderate loads (70–85% of one repetition maximum: 1RM) and volumes (2–3 sets per exercise) [81,87]. Rest periods should last 1–2 min, though some encourage shorter rests to evoke endogenous anabolic hormone secretion [87,96,97,98]. In terms of exercise selection for older adults, multi-joint exercises have more functional relevance [99]. Yet, unilateral exercises should not be discouraged. Machine-based exercises are recommended over free weights for those new to weight training since they require less skill and coordination and are therefore thought to be safer. One to two exercises per muscle group are adequate for persons new to resistance exercise [99].

In terms of the rigor of weight training geared toward older adults and those with sarcopenia, rates of perceived exertion, which are a subjective measure of exercise intensity, should initially fall within a range of 3–5 (based on the 10-point Borg scale) and progress to 6–8 over time [87]. Gradual increases in exercise loads in excess of 80% of a person’s 1RM is an attainable goal for weight training novices [100,101,102,103]. Yet, loads equal to 65–75% of an older person’s 1RM load will increase their strength and may be preferable since it also limits their injury risk [99]. At 80% of 1RM loads, the number of repetitions performed to momentary failure generally falls between 8–15 [96]. The number of repetitions needed to reach failure for machines is slightly higher than for free weights, presumably because the latter requires more muscle recruitment for stabilization and balance [96]. Weight training programs for older adults should also consider high-speed power exercises where concentric repetition phases occur rapidly against the light (40–60% of 1RM) loads [81]. Power exercises are most beneficial for type II muscle fibers which see the most atrophy with aging and sarcopenia.

In support of the aforementioned exercise prescription guidelines, Bagheri showed that in older sarcopenic men, an exercise frequency of three times weekly, with 1–3 sets per muscle group and workout, and 6–12 repetitions per set effectively induced strength gains [104]. With a randomized trial of older men, Teodoro et al. concluded that similar improvements occurred with concentric repetitions performed to volitional fatigue [105]. Muscle hypertrophy occurs more easily in the general population than in those with sarcopenia. Thus, strength gains are thought as a better index to assess resistive exercise progress in sarcopenic patients. After a 12-month program administered to older adults with no crossover, Yoshiko et al. showed an ~16% gain in knee extensor torque with low (20–40% 1RM) loads while a control group decreased by 21% [106]. Since both aerobic and resistive training are recommended to improve the health of older patients and those with sarcopenia, Bagheri et al. examined training orders with both exercise modalities [104]. Results showed that significantly more strength was gained if a resistance exercise was performed after endurance training, as compared to when the order was reversed [104].

Studies also examined relationships between muscle size and function in older adults and those with sarcopenia. Muscle quantity was assessed with mid-thigh CT scans in 214 patients [20]. In addition to CT, they underwent other tests to assess muscle function and mass [20]. Knee extensor strength correlated significantly (r = 0.60) with thigh cross-sectional area [20]. It was concluded that CT was useful to assess muscle function and sarcopenia [20]. A related study examined MRI indices as sarcopenia correlates in women (n = 26; 81 ± 8 years of age) [107]. Results showed strong correlations between anthropometry and MRI indices. Data from older women could help identify those at risk of developing sarcopenia at younger ages and identify it at earlier stages [107].

A systematic review and meta-analysis on resistance exercise for older sarcopenic persons revealed strength gains were more common than those for muscle mass [108]. Whole muscle hypertrophy measurements sometimes show gains in sarcopenic patients due to their low starting muscle mass [32,109,110]. Yet, research shows older adults can experience hypertrophy (~30% size increase after 16 weeks of resistance training), fiber type transitions (from type IIx to IIa), and incorporate new nuclei into muscle cells [111]. Among older men with sarcopenia, greater hypertrophy occurs with concurrent protein supplementation and moderate-to-heavy resistance exercise (>65% 1 RM) [112,113]. Weight training led to muscle hypertrophy after 24 weeks concurrent to modest protein supplementation, which elicited a 4.6% thigh muscle cross-sectional area gain in mobility-limited older adults [100]. Protein supplementation may be most beneficial to older adults and those with sarcopenia since their diets typically have insufficient energy and protein [30]. A 2019 study by Vikberg et al. examined a periodized whole-body resistance training and protein supplementation program on muscle mass and strength changes in older pre-sarcopenic subjects [110]. Resistance training entailed 10 repetition sets at a moderate intensity while post-workout protein supplementation (21 g per workout) occurred after each workout [110]. After ten weeks, lean body mass incresed by 1.17 kg [110]. Stec and colleagues examined training responses to workouts in older adults with muscle atrophy at different exercise intensities and training frequencies [112]. Their diets received protein supplementation at a dose equal to 0.3 g·kg^−1^ [112]. Results showed that alternate workouts with high- or low-load resistance training three days per week yielded the best gains [112]. Evidence suggests that resistive exercise combined with a high protein diet that may include supplementation best reduces the risk of sarcopenia in older adults [110,112,113].

## 4. Discussion

This review discusses sarcopenia and how it may be mitigated through diet and exercise. Dietary and exercise guidelines for older people should be different from those in the public, particularly for those with sarcopenia. It was previously thought that older persons with sarcopenia, osteoporosis, or other comorbidities could not tolerate high-intensity resistance training. While sarcopenia reduces muscle mass, older people may consistently improve their strength if they routinely engage in resistance exercise. A strategy to limit sarcopenia should combine diet and exercise. Results sighted in this review suggest that while diet and exercise interventions administered individually limit the occurrence of sarcopenia, combining the treatments is the most effective way to combat this condition [30,100,110,112,113,114].

Sarcopenia sees negative intracellular protein turnover that elicits muscle atrophy and increases the likelihood of clinical conditions that undermine the quality of life [30]. Resistance exercise alone does not lead to net MPS and hypertrophy until all EAA, whether consumed through animal sources, food combining, or supplementation, are provided [30]. In a similar fashion, dietary protein alone may induce a positive intracellular protein balance even without resistance exercise, though this anabolism is transient and fleeting and causes an increase in MPS without a change in its rate of breakdown [30]. Yet, if it is concurrent with resistance exercise, intracellular MPS is increased and prolonged. Thus, combining resistance exercise with a high protein intake appears best to mitigate sarcopenia [30]. However, not all studies advocate for combining these treatments [100].

Mobility-impaired older adults (n = 80) were subdivided into two groups to examine the effects of whey protein supplementation on muscle mass, strength, and physical function as part of a double-blind study [100]. With no crossover, each group completed a six-month progressive high-intensity resistance exercise intervention [100]. Subjects were randomized to either a whey protein dietary supplement with 20 g of protein per serving or an isocaloric maltodextrin solution that served as a control. Subjects in each group ingested two servings per day and nutritional intake was monitored with two three-day food logs at the start and end of the six-month intervention. Inter-group muscle mass, strength, and physical function differences saw greater gains from whey protein supplementation, yet these changes were not significant. Results implied that whey protein did not offer any extra benefit beyond that provided by resistance exercise [100].

Unlike the prior study [100], other investigations saw benefits from combined protein-resistive exercise interventions [110,112,113]. To examine a combined protein-resistance exercise intervention in older pre-sarcopenic adults, subjects were divided into two groups with no crossover for ten weeks [110]. One group underwent a nutrition-resistance exercise intervention (n = 36) while another received neither treatment (n = 34). Resistance exercise was conducted three times weekly and aimed to reach a perceived exertion value of 6–7 on the 10-point Borg scale. Subjects in that same group had the option to consume milk-based dietary supplements with 21–30 g of protein [110]. The protein-resistance exercise group saw significant improvements in muscle mass and body composition as well as on a performance test over time. It was concluded that the same treatment maintained functional strength, muscle mass, and body composition in older pre-sarcopenic adults [110].

A four-group trial sought to optimize resistance exercise prescriptions for older adults with muscle atrophy [112]. Each group had training loads and frequencies prescribed in accordance with their group assignment. Subjects (n = 64) did the same four weeks of pre-training exercises three times per week, followed by 30 weeks of their group’s training protocol [112]. In addition to exercise, all subjects consumed a whey protein supplement with 0.6 g whey protein·kg body weight^−1^ over the 30-week intervention [112]. Over time, all subjects exhibited thigh and total muscle mass gains, with the greatest benefits observed in those who performed two high-load and one low-load resistance exercise workouts per week. It was concluded older adults benefit from resistance exercise performed twice per week, with greater results from an additional weekly low-load workout comprised repetitions performed at high velocities [112].

Osteosarcopenia is a condition comprised both osteopenia and sarcopenia. In older men (n = 43). the effects of resistance exercise were assessed over 28 a week period [113]. With no crossover, osteosarcopenic subjects were divided into two groups: those who underwent a nutritional-resistance exercise intervention and those who remained untrained and served as controls. Both groups consumed protein (up to 1.5 g·day^−1^ for the experimental group, and 1.2 g·day^−1^ for controls) and vitamin D (up to 800 IE·day^−1^) supplements [113]. Results show the experimental group had significant inter-group improvements in skeletal muscle mass and handgrip strength. There were no adverse effects from dietary supplementation or resistance exercise. The nutritional-resistance exercise intervention was deemed a good training modality for older men [113].

## 5. Conclusions

Dietary and exercise guidance for older people should differ from those for the general public, particularly if they have sarcopenia. Currently, a more holistic approach to diet and exercise reduces the likelihood sarcopenia. More succinct and age-appropriate guidelines need to be developed and disseminated so older adults ingest appropriate amounts of all nutrients throughout their lifespan. This issue is perhaps best illustrated by protein, whereby sedentary healthy young adults are encouraged to consume the RDA of 0.8 g·kg^−1^; yet, an older person may have to ingest over twice (1.8 g·kg^−1^) that amount, in 25–30 g feedings, to maintain muscle mass [30,44]. Aside from sufficient energy and macro- and micronutrient intakes, HMB is a dietary aide that warrants consideration to limit the occurrence and severity of sarcopenia since it showed promise for persons with muscle wasting without side effects or changes to diet and exercise habits [72,73]. More longitudinal studies are needed to ensure its minimal side effects but HMB could serve as a useful supplement for healthy persons and those with sarcopenia [72,73]. Regarding lifestyle modifications, only 8.7–10% of older Americans participate in resistance exercise, which is thought ideal to treat sarcopenia [2,81]. Evidence of an optimal resistance exercise prescription for sarcopenic patients is limited [87]. Resistance exercise program design may be even more important for older adults as factors such as health/family histories, injuries, and pre-existing fitness levels should be considered [94]. General resistance exercise guidelines for older adults include 2–3 workouts per week against moderate loads and volumes [81,87]. Sarcopenic patients, due to reduced muscle mass, fatigue easily and thus have a low workout tolerance. To accommodate for their fatiguability, perceived exertion values for sarcopenia patients should initially fall between 3–5 on the 10-point Borg scale and progress to 6–8 over time [87]. The diet and exercise guidelines provided in this paper offer a more holistic approach, devoid of pharmaceutical treatments, that act as a preventive measure to mitigate the likelihood and severity of sarcopenia.

## References

[1] Rosenberg I.H. (1997). Sarcopenia: Origins and clinical relevance. J. Nutr..

[2] Oliveira C.L.P., Dionne I.J., Prado C.M. (2018). Are Canadian protein and physical activity guidelines optimal for sarcopenia prevention in older adults?. Appl. Physiol. Nutr. Metab..

[3] Cruz-Jentoft A.J., Bahat G., Bauer J., Boirie Y., Bruyère O., Cederholm T., Cooper C., Landi F., Rolland Y., Sayer A.A. (2019). Sarcopenia: Revised European consensus on definition and diagnosis. Age Ageing.

[4] Da Silva T.L., dos Santos Chiapetta Salgado Nogueira V., Mulder A.P. (2021). Sarcopenia and poor muscle quality associated with sever obesity in young adults and middle-aged adults. Clin. Nutr..

[5] Koo K.B. (2022). Assessment of muscle quantity, quality and function. J. Obes. Metab. Syndr..

[6] Maykish A., Sikalidis A.K. (2020). Utilization of hydroxyl-methyl butyrate, leucine, glutamine and arginine supplementation in nutritional management of sarcopenia-implication and clinical considerations for type 2 diabetes mellitus risk modulation. J. Pers. Med..

[7] Xie L., Jiang J., Fu H., Zhang W., Yang L., Yang M. (2022). Malnutrition in relation to muscle mass, muscle quality, and muscle strength in hospitalized older adults. J. Am. Med. Dir. Assoc..

[8] Hickson M. (2015). Nutritional interventions in sarcopenia: A critical review. Proc. Nutr. Soc..

[9] Ahn H., Kim D.W., Ko Y., Ha J., Shin Y.B., Lee J., Sung Y.S., Kim K.W. (2021). Updated systematic review and meta-analysis on diagnostic issues and the prognostic impact of myosteatosis: A new paradigm beyond sarcopenia. Ageing Res. Rev..

[10] Lattanzi B., Nardelli S., Pigliacelli A., Di Cola S., Farcomeni A., D’Ambrosio D., Gioia S., Corradini S.G., Lucidi C., Mennini G. (2019). The additive value of sarcopenia, myosteatosis and hepatic encephalopathy in the predictivity of model for end-stage liver disease. Dig. Liver Dis..

[11] Ghiotto L., Muollo V., Tatangelo T., Schena F., Rosi A.P. (2022). Exercise and physical performance in older adults with sarcopenic obesity: A systematic review. Front. Endorcrinol..

[12] Harada K., Suzuki S., Ishii H., Aoki T., Hirayama K., Shibata Y., Negishi Y., Sumi T., Kawashima K., Kunimara A. (2017). Impact of Skeletal muscle mass on long-term adverse cardiovascular outcomes in patients with chronic kidney disease. Am. J. Cardiol..

[13] Kim J.H., Lim S., Choi S.H., Kim K.M., Yoon J.W., Kim K.W., Lim J.Y., Park K.S., Jang H.C. (2014). Sarcopenia: An independent predictor of mortality in community-dwelling older Korean men. J. Gerontol. A Biomed. Med. Sci..

[14] Kakehi S., Wakabayashi H., Inuma H., Inose T., Shioya M., Aoyama Y., Hara T., Uchimura K., Tomita K., Okamoto M. (2022). Rehabilitation nutrition and exercise therapy for sarcopenia. World J. Mens Health.

[15] Yoo S.Z., No M.H., Heo J.W., Park D.H., Kang J.H., Kim S.H., Kwas H.B. (2018). Role of exercise in age-related sarcopenia. J. Exerc. Rehabil..

[16] Chen L.K., Woo J., Assantachai P., Auyeung T.W., Chou M.Y., Iijima K., Jang H.C., Kang L., Kim M., Kim S. (2020). Asian Working Group for Sarcopenia: 2019 consensus update on sarcopenia diagnosis and treatment. J. Am. Med. Dir. Assoc..

[17] Kimura K., Chang X.W., Inoue A., Hu L., Koike T., Kuzuya M. (2014). Beta-hydroxy-beta-methylbutyrate facilitates PI3K/Akt-dependent mammalian target of rapamyacin and Fox01/3a phosphorylations and alleviates tumor necrosis factor alpha/interferon gamma-induced MuRF-1 expression in C2C12 cells. Nutr. Res..

[18] Fielding R.A., Vellas B., Evans W.J., Bhasin S., Morley J.E., Newman A.B., van Kan G.A., Andrieu S., Bauer J., Breuille D. (2011). Sarcopenia: An undiagnosed condition in older adults: Current consensus definition: Prevalence, etiology, and consequences. International Working Group on Sarcopenia. J. Am. Med. Dir. Assoc..

[19] Marty E., Liu Y., Samuel A., Or O., Lane J. (2017). A review of sarcopenia: Enhancing awareness of an increasingly prevalent disease. Bone.

[20] Oba H., Matsui Y., Arai H., Watanabe T., Iida H., Mizuno T., Yamashita S., Ishizuka S., Suzuki Y., Hiraiwa H. (2021). Evaluation of muscle quality and quantity for assessment of sarcopenia using mid-thigh computed tomography: A cohort study. BMC Geriatr..

[21] Barbosa-Silva T.G., Menezes A.M., Bielemann R.M., Malmstrom T.K., Gonzalez M.C. (2016). Grupo de Estudos em Composição Corporal e Nutrição (COCONUT). Enhancing SARC-F: Improving sarcopenia screening in the clinical practice. J. Am. Med. Dir. Assoc..

[22] Harris T. (1997). Muscle mass and strength: Relation to function in population studies. J. Nutr..

[23] Larsen B.A., Wassel C.L., Kritchevsky S.B., Strotmeyer E.S., Criqui M.H., Kanaya A.M., Fried L.F., Schwartz A.V., Harris T.B., Ix J.H. (2016). Association of muscle mass, area, and strength with incident diabetes in older adults: The Health ABC Study. J. Clin. Endocrinol. Metab..

[24] Marzetti E., Calvani R., Tosato M., Cesari M., Di Bari M., Cherubini A., Collamati A., D’Angelo E., Pahor M., Bernabei R. (2017). Sarcopenia: An overview. Ageing Clin. Exp. Res..

[25] Choi K.M. (2016). Sarcopenia and sarcopenic obesity. Korean J. Intern. Med..

[26] Keller K., Engelhardt M. (2014). Strength and muscle mass loss with aging process. Age and strength loss. Muscles Ligaments Tendons J..

[27] Baumgartner R.N., Koehler K.M., Gallagher D., Romero L., Heymsfield S.B., Ross R.R., Garry P.J., Lindeman R.D. (1998). Epidemiology of sarcopenia among the elderly in New Mexico. Am. J. Epidemiol..

[28] Von Haehling S., Morley J.E., Anjer S.D. (2010). An overview of sarcopenia: Facts and numbers on prevalence and clinical impact. J. Cachexia Sarcopenia Muscle.

[29] Shafiee G., Keshtkar A., Soltani A., Ahadi Z., Larijani B., Heshmat R. (2017). Prevalence of sarcopenia in the world: A systematic review and meta-analysis of general population studies. J. Diabetes Metab. Disord..

[30] Kim I.-Y., Park S., Jang J., Wolfe R.R. (2020). Understanding Muscle Protein Dynamics: Technical Considerations for Advancing Sarcopenia Research. Ann. Geriatr. Med. Res..

[31] Ganapathy A., Nieves J.W. (2020). Nutrition and Sarcopenia—What Do We Know?. Nutrients.

[32] Deldicque L. (2020). Protein Intake and Exercise-Induced Skeletal Muscle Hypertrophy: An Update. Nutrients.

[33] Stein T.P., Leskiw M.J., Schluter M.D., Hoyt R.W., Lane H.W., Gretebeck R.E., LeBlanc A. (1999). Energy expenditure and balance during spaceflight on the space shuttle. Am. J. Physiol. Integr. Comp. Physiol..

[34] Stein T. (2000). The relationship between dietary intake, exercise, energy balance and the space craft environment. Pflügers Arch..

[35] Fulgoni V.L. (2008). Current protein intake in America: Analysis of the National Health and Nutrition Examination Survey, 2003–2004. Am. J. Clin. Nutr..

[36] Volpi E., Campbell W.W., Dwyer J.T., Johnson M.A., Jensen G.L., Morley J.E., Wolfe R.R. (2012). Is the Optimal Level of Protein Intake for Older Adults Greater Than the Recommended Dietary Allowance?. J. Gerontol. A.

[37] Bauer J., Biolo G., Cederholm T., Cesari M., Cruz-Jentoft A.J., Morley J.E., Phillips S., Sieber C., Stehle P., Teta D. (2013). Evidence-Based Recommendations for Optimal Dietary Protein Intake in Older People: A Position Paper From the PROT-AGE Study Group. J. Am. Med. Dir. Assoc..

[38] Gray-Donald K., Arnaud-McKenzie D.S., Gaudreau P., Morais J.A., Shatenstein B., Payette H. (2014). Protein Intake Protects against Weight Loss in Healthy Community-Dwelling Older Adults. J. Nutr..

[39] Koopman R. (2010). Dietary protein and exercise training in ageing. Proc. Nutr. Soc..

[40] Houston D.K., Nicklas B.J., Ding J., Harris T.B., Tylavsky F.A., Newman A.B., Lee J.S., Sahyoun N.R., Visser M., Kritchevsky S.B. (2008). Dietary protein intake is associated with lean mass change in older, community-dwelling adults: The Health, Aging, and Body Composition (Health ABC) Study. Am. J. Clin. Nutr..

[41] Beasley J.M., Wertheim B.C., LaCroix A.Z., Prentice R.L., Neuhouser M.L., Tinker L.F., Kritchevsky S., Shikany J.M., Eaton C., Chen Z. (2013). Biomarker-Calibrated Protein Intake and Physical Function in the Women’s Health Initiative. J. Am. Geriatr. Soc..

[42] Naseeb M.A., Volpe S.L. (2017). Protein and exercise in the prevention of sarcopenia and aging. Nutr. Res..

[43] Deutz N.E., Bauer J.M., Barazzoni R., Biolo G., Boirie Y., Bosy-Westphal A., Cederholm T., Cruz-Jentoft A., Krznariç Z., Nair K.S. (2014). Protein intake and exercise for optimal muscle function with aging: Recommendations from the ESPEN Expert Group. Clin. Nutr..

[44] Paddon-Jones D., Rasmussen B.B. (2009). Dietary protein recommendations and the prevention of sarcopenia. Curr. Opin. Clin. Nutr. Metab. Care.

[45] Cuthbertson D., Smith K., Babraj J., Leese G., Waddell T., Atherton P., Wackerhage H., Taylor P.M., Rennie M.J. (2004). Anabolic signaling deficits underlie amino acid resistance of wasting, aging muscle. FASEB J..

[46] Volpi E., Mittendorfer B., Rasmussen B.B., Wolfe R.R. (2000). The Response of Muscle Protein Anabolism to Combined Hyperaminoacidemia and Glucose-Induced Hyperinsulinemia Is Impaired in the Elderly. J. Clin. Endocrinol. Metab..

[47] Symons T.B., E Schutzler S., Cocke T.L., Chinkes D.L., Wolfe R.R., Paddon-Jones D. (2007). Aging does not impair the anabolic response to a protein-rich meal. Am. J. Clin. Nutr..

[48] Lo J.H.-T., U K.P., Yiu T., Ong M.T.-Y., Lee W.Y.-W. (2020). Sarcopenia: Current treatments and new regenerative therapeutic approaches. J. Orthop. Transl..

[49] Fujita S., Rasmussen B.B., Cadenas J.G., Drummond M.J., Glynn E.L., Sattler F.R., Volpi E. (2007). Aerobic Exercise Overcomes the Age-Related Insulin Resistance of Muscle Protein Metabolism by Improving Endothelial Function and Akt/Mammalian Target of Rapamycin Signaling. Diabetes.

[50] Katsanos C.S., Kobayashi H., Sheffield-Moore M., Aarsland A., Wolfe R.R. (2005). Aging is associated with diminished accretion of muscle proteins after the ingestion of a small bolus of essential amino acids. Am. J. Clin. Nutr..

[51] Yang Y., Breen L., Burd N.A., Hector A.J., Churchward-Venne T.A., Josse A.R., Tarnopolsky M.A., Phillips S.M. (2012). Resistance exercise enhances myofibrillar protein synthesis with graded intakes of whey protein in older men. Br. J. Nutr..

[52] Katsanos C.S., Kobayashi H., Sheffield-Moore M., Aarsland A., Wolfe R.R. (2006). A high proportion of leucine is required for optimal stimulation of the rate of muscle protein synthesis by essential amino acids in the elderly. Am. J. Physiol. Metab..

[53] Ferrando A.A., Paddon-Jones D., Hays N.P., Kortebein P., Ronsen O., Williams R.H., McComb A., Symons T.B., Wolfe R.R., Evans W. (2010). EAA supplementation to increase nitrogen intake improves muscle function during bed rest in the elderly. Clin. Nutr..

[54] Phillips S.M., Martinson W. (2018). Nutrient-rich, high-quality, protein-containing dairy foods in combination with exercise in aging persons to mitigate sarcopenia. Nutr. Rev..

[55] Lin C.-C., Yeh S.-L. (2022). Reply to the letter to the editor: Effects of adequate dietary protein with whey protein, leucine, and vitamin D supplementation on sarcopenia in older adults: An open-label, parallel-group study. Clin. Nutr..

[56] Volpi E., Kobayashi H., Sheffield-Moore M., Mittendorfer B., Wolfe R.R. (2003). Essential amino acids are primarily responsible for the amino acid stimulation of muscle protein anabolism in healthy elderly adults. Am. J. Clin. Nutr..

[57] Biolo G., De Cicco M., Mas V.D., Lorenzon S., Antonione R., Ciocchi B., Barazzoni R., Zanetti M., Dore F., Guarnieri G. (2006). Response of muscle protein and glutamine kinetics to branched-chain–enriched amino acids in intensive care patients after radical cancer surgery. Nutrition.

[58] Marchesini G., Zoli M., Dondi C., Bianchi G., Cirulli M., Pisi E. (1982). Anticatabolic Effect of Branched-Chain Amino Acid-Enriched Solutions in Patients with Liver Cirrhosis. Hepatology.

[59] Kim H.K., Suzuki T., Saito K., Yoshida H., Kobayashi H., Kato H., Katayama M. (2011). Effects of Exercise and Amino Acid Supplementation on Body Composition and Physical Function in Community-Dwelling Elderly Japanese Sarcopenic Women: A Randomized Controlled Trial. J. Am. Geriatr. Soc..

[60] Børsheim E., Bui Q.-U.T., Tissier S., Kobayashi H., Ferrando A.A., Wolfe R.R. (2008). Effect of amino acid supplementation on muscle mass, strength and physical function in elderly. Clin. Nutr..

[61] Verhoeven S., Vanschoonbeek K., Verdijk L.B., Koopman R., Wodzig W.K., Dendale P., van Loon L.J. (2009). Long-term leucine supplementation does not increase muscle mass or strength in healthy elderly men. Am. J. Clin. Nutr..

[62] Leenders M., Verdijk L.B., van der Hoeven L., van Kranenburg J., Hartgens F., Wodzig W.K.W.H., Saris W.H.M., van Loon L.J.C. (2011). Prolonged Leucine Supplementation Does Not Augment Muscle Mass or Affect Glycemic Control in Elderly Type 2 Diabetic Men. J. Nutr..

[63] Kumar P., Umakanth S., Girish N. (2022). A review of the components of exercise prescription for sarcopenic older adults. Eur. Geriatr. Med..

[64] Moore S.A., Hrisos N., Errington L., Rochester L., Rodgers H., Witham M., Sayer A.A. (2020). Exercise as a treatment for sarcopenia: An umbrella review of systematic review evidence. Physiotherapy.

[65] Gkekas N.K., Anagnostis P., Paraschou V., Stamiris D., Dellis S., Kenanidis E., Potoupnis M., Tsiridis E., Goulis D.G. (2021). The effect of vitamin D plus protein supplementation on sarcopenia: A systematic review and meta-analysis of randomized controlled trials. Maturitas.

[66] Bell K.E., Snijders T., Zulyniak M., Kumbhare D., Parise G., Chabowski A., Phillips S.M. (2017). A whey protein-based multi-ingredient nutritional supplement stimulates gains in lean body mass and strength in healthy older men: A randomized controlled trial. PLoS ONE.

[67] Uchitomi R., Oyabu M., Kamei Y. (2020). Vitamin D and Sarcopenia: Potential of Vitamin D Supplementation in Sarcopenia Prevention and Treatment. Nutrients.

[68] Forrest K.Y., Stuhldreher W.L. (2011). Prevalence and correlates of vitamin D deficiency in US adults. Nutr. Res..

[69] Garcia M., Seelaender M., Sotiropoulos A., Coletti D., Lancha A.H. (2019). Vitamin D, muscle recovery, sarcopenia, cachexia, and muscle atrophy. Nutrition.

[70] Cruz-Jentoft A.J., Morley J.E. (2012). Sarcopenia.

[71] Tieland M., Trouwborst I., Clark B.C. (2017). Skeletal muscle performance and ageing. J. Cachexia Sarcopenia Muscle.

[72] Holecek M., Muthny T., Kovarik M., Sispera L. (2009). Effect of beta-hydroxy-beta-methylbutyrate (HMB) on protein metabolism in whole body and in selected tissues. Food Chem. Toxicol..

[73] Holeček M. (2017). Beta-hydroxy-beta-methylbutyrate supplementation and skeletal muscle in healthy and muscle-wasting conditions. J. Cachexia Sarcopenia Muscle.

[74] Kamei Y., Hatazawa Y., Uchitomi R., Yoshimura R., Miura S. (2020). Regulation of Skeletal Muscle Function by Amino Acids. Nutrients.

[75] Prado C.M., Orsso C.E., Pereira S.L., Atherton P.J., Deutz N.E.P. (2022). Effects of β-hydroxy β-methylbutyrate (HMB) supplementation on muscle mass, function, and other outcomes in patients with cancer: A systematic review. J. Cachexia Sarcopenia Muscle.

[76] Solerte S.B., Gazzaruso C., Bonacasa R., Rondanelli M., Zamboni M., Basso C., Locatelli E., Schifino N., Giustina A., Fioravanti M. (2008). Nutritional Supplements with Oral Amino Acid Mixtures Increases Whole-Body Lean Mass and Insulin Sensitivity in Elderly Subjects with Sarcopenia. Am. J. Cardiol..

[77] Hsieh L.C., Chien S.L., Huang S., Tseng H.F., Chang C.K. (2006). Anti-inflammatory and anticatabolic effects of short-term β-hyroxy- β-methylbutyrate supplementation on chronic obstructive pulmonary disease patients in intensive care unit. Asia Pac. J. Clin. Nutr..

[78] Fukagawa N.K. (2013). Protein and amino acid supplementation in older humans. Amino Acids.

[79] Martone A.M., Bianchi L., Abete P., Bellelli G., Bo M., Cherubini A., Corica F., Di Bari M., Maggio M., Manca G.M. (2017). The incidence of sarcopenia among hospitalized older patients: Results from the Glisten study. J. Cachexia Sarcopenia Muscle.

[80] Landi F., Marzetti E., Martone A.M., Bernabei R., Onder G. (2013). Exercise as a remedy for sarcopenia. Curr. Opin. Clin. Nutr. Metab. Care.

[81] Fragala M.S., Cadore E.L., Dorgo S., Izquierdo M., Kraemer W.J., Peterson M.D., Ryan E.D. (2019). Resistance Training for Older Adults. J. Strength Cond. Res..

[82] Shen Y., Shi Q., Nong K., Li S., Yue J., Huang J., Dong B., Beauchamp M., Hao Q. (2023). Exercise for sarcopenia in older people: A systematic review and network meta-analysis. J. Cachexia Sarcopenia Muscle.

[83] Elgaddal N., Kramarow E.A., Reuben C. (2022). Physical Activity Among Adults Aged 18 and Over: United States, 2020.

[84] Robinson M.M., Dasari S., Konopka A.R., Johnson M.L., Manjunatha S., Esponda R.R., Carter R.E., Lanza I.R., Nair K.S. (2017). Enhanced protein translation underlies improved metabolic and physical adaptations to different exercise training modes in young and old humans. Cell Metab..

[85] Orr R., de Vos N.J., Singh N.A., Ross D.A., Stavrinos T.M., Fiatarone-Singh M.A. (2006). Power Training Improves Balance in Healthy Older Adults. J. Gerontol. Ser. A.

[86] Oliveira J.S., Pinheiro M.B., Fairhall N., Walsh S., Franks T.C., Kwok W., Bauman A., Sherrington C. (2020). Evidence on Physical Activity and the Prevention of Frailty and Sarcopenia Among Older People: A Systematic Review to Inform the World Health Organization Physical Activity Guidelines. J. Phys. Act. Health.

[87] Hurst C., Robinson S.M., Witham M.D., Dodds R.M., Granic A., Buckland C., De Biase S., Finnegan S., Rochester L., A Skelton D. (2022). Resistance exercise as a treatment for sarcopenia: Prescription and delivery. Age Ageing.

[88] Fiatarone M.A., Marks E.C., Ryan N.D., Meredith C.N., Lipsitz L.A., Evans W.J. (1990). High-intensity strength training in nonagenarians: Effects on skeletal muscle. JAMA.

[89] Maruya K., Asakawa Y., Ishibashi H., Fujita H., Arai T., Yamaguchi H. (2016). Effect of a simple and adherent home exercise program on the physical function of community dwelling adults sixty years of age and older with pre-sarcopenia or sarcopenia. J. Phys. Ther. Sci..

[90] Keller K. (2018). Sarcopenia. Wien. Med. Wochenschr..

[91] Bauer J., Morley J.E., Schols A.M.W.J., Ferrucci L., Cruz-Jentoft A.J., Dent E., Baracos V.E., Crawford J.A., Doehner W., Heymsfield S.B. (2019). Sarcopenia: A Time for Action. An SCWD Position Paper. J. Cachexia Sarcopenia Muscle.

[92] Dhillon R.J., Hasni S. (2017). Pathogenesis and Management of Sarcopenia. Clin. Geriatr. Med..

[93] Giallauria F., Cittadini A., Smart N.A., Vigorito C. (2016). Resistance training and sarcopenia. Monaldi Arch. Chest Dis..

[94] Ratamess N.A., Alvar B.A., Evetoch T.E., Housh T.J., Ben Kibler W., Kraemer W.J., Triplett N.T. (2009). Progression models in resistance training for healthy adults. Med. Sci. Sports Exerc..

[95] Kraemer W.J., Fragala M.S. (2006). Personalize it: Program design in resistance training. ACSMs Health Fit. J..

[96] Law T.D., Clark L.A., Clark B.C. (2016). Resistance Exercise to Prevent and Manage Sarcopenia and Dynapenia. Annu. Rev. Gerontol. Geriatr..

[97] De Salles B.F., Simão R., Miranda F., da Silva Novaes J., Lemos A., Willardson J.M. (2009). Rest Interval between Sets in Strength Training. Sports Med..

[98] Willardson J.M. (2006). A Brief Review: Factors Affecting the Length of the Rest Interval Between Resistance Exercise Sets. J. Strength Cond. Res..

[99] Willoughby D.S. (2015). Current Comments are official statements by the American College of Sports Medicine concerning topics of interest to the public at large. Resistance Training and the Older Adults.

[100] Chalé A., Cloutier G.J., Hau C., Phillips E.M., Dallal G.E., Fielding R.A. (2013). Efficacy of Whey Protein Supplementation on Resistance Exercise–Induced Changes in Lean Mass, Muscle Strength, and Physical Function in Mobility-Limited Older Adults. J. Gerontol. A Biol. Sci. Med. Sci..

[101] Fiatarone M.A., O’Neill E.F., Ryan N.D., Clements K.M., Solares G.R., Nelson M.E., Roberts S.B., Kehayias J.J., Lipsitz L.A., Evans W.J. (1994). Exercise Training and Nutritional Supplementation for Physical Frailty in Very Elderly People. N. Engl. J. Med..

[102] Reeves N.D., Maganaris C.N., Narici M.V. (2003). Effect of strength training on human patella tendon mechanical properties of older individuals. J. Physiol..

[103] Singh N.A., Quine S., Clemson L.M., Williams E.J., Williamson D.A., Stavrinos T.M., Grady J.N., Perry T.J., Lloyd B.D., Smith E.U. (2012). Effects of High-Intensity Progressive Resistance Training and Targeted Multidisciplinary Treatment of Frailty on Mortality and Nursing Home Admissions after Hip Fracture: A Randomized Controlled Trial. J. Am. Med. Dir. Assoc..

[104] Bagheri R., Moghadam B.H., Church D.D., Tinsley G.M., Eskandari M., Moghadam B.H., Motevalli M.S., Baker J.S., Robergs R.A., Wong A. (2020). The effects of concurrent training order on body composition and serum concentrations of follistatin, myostatin and GDF11 in sarcopenic elderly men. Exp. Gerontol..

[105] Teodoro J.L., Izquierdo M., da Silva L.X., Baroni B.M., Grazioli R., Lopez P., Fritsch C.G., Radaelli R., de Asteasu M.L.S., Bottaro M. (2020). Effects of long-term concurrent training to failure or not in muscle power output, muscle quality and cardiometabolic risk factors in older men: A secondary analysis of a randomized clinical trial. Exp. Gerontol..

[106] Yoshiko A., Kaji T., Sugiyama H., Koike T., Oshida Y., Akima H. (2017). Effect of 12-month resistance and endurance training on quality, quantity, and function of skeletal muscle in older adults requiring long-term care. Exp. Gerontol..

[107] Sanz-Requena R., Martínez-Arnau F.M., Pablos-Monzó A., Flor-Rufino C., Barrachina-Igual J., García-Martí G., Martí-Bonmatí L., Pérez-Ros P. (2020). The Role of Imaging Biomarkers in the Assessment of Sarcopenia. Diagnostics.

[108] Bao W., Sun Y., Zhang T., Zou L., Wu X., Wang D., Chen Z. (2020). Exercise Programs for Muscle Mass, Muscle Strength and Physical Performance in Older Adults with Sarcopenia: A Systematic Review and Meta-Analysis. Aging Dis..

[109] Kemmler W., Kohl M., Fröhlich M., Jakob F., Engelke K., Stengel S., Schoene D. (2020). Effects of High-Intensity Resistance Training on Osteopenia and Sarcopenia Parameters in Older Men with Osteosarcopenia—One-Year Results of the Randomized Controlled Franconian Osteopenia and Sarcopenia Trial (FrOST). J. Bone Miner. Res..

[110] Vikberg S., Sorlen N., Branden L., Johansson J., Nordström A., Hult A., Nordström P. (2019). Effects of resistance training on functional strength and muscle mass in 70-year-old individuals with pre-sarcopenia: A randomized controlled trial. J. Am. Med. Dir. Assoc..

[111] Hikida R.S., Staron R.S., Hagerman F.C., Walsh S., Kaiser E., Shell S., Hervey S. (2000). Effects of High-Intensity Resistance Training on Untrained Older Men. II. Muscle Fiber Characteristics and Nucleo-Cytoplasmic Relationships. J. Gerontol. Ser. A.

[112] Stec M.J., Thalacker-Mercer A., Mayhew D.L., Kelly N.A., Tuggle S.C., Merritt E.K., Brown C.J., Windham S.T., Dell’Italia L.J., Bickel C.S. (2017). Randomized, four-arm, dose-response clinical trial to optimize resistance exercise training for older adults with age-related muscle atrophy. Exp. Gerontol..

[113] Lichtenberg T., von Stengel S., Sieber C., Kemmler W. (2019). The Favorable Effects of a High-Intensity Resistance Training on Sarcopenia in Older Community-Dwelling Men with Osteosarcopenia: The Randomized Controlled FrOST Study. Clin. Interv. Aging.

[114] Kirwan R., McCullough D., Butler T., de Heredia F.P., Davies I.G., Stewart C. (2020). Sarcopenia during COVID-19 lockdown restrictions: Long-term health effects of short-term muscle loss. GeroScience.

